# *CalQuo*^*2*^: Automated Fourier-space, population-level quantification of global intracellular calcium responses

**DOI:** 10.1038/s41598-017-05322-z

**Published:** 2017-07-14

**Authors:** Angela M. Lee, Huw Colin-York, Marco Fritzsche

**Affiliations:** 1 MRC Human Immunology Unit, Weatherall Institute of Molecular Medicine, University of Oxford, Headley Way, Oxford, OX3 9DS United Kingdom; 2 Kennedy Institute for Rheumatology, Roosevelt Drive, University of Oxford, Oxford, OX3 7FY United Kingdom

## Abstract

Intracellular calcium acts as a secondary messenger in a wide variety of crucial biological signaling processes. Advances in fluorescence microscopy and calcium sensitive dyes has led to the routine quantification of calcium responses in non-excitable cells. However, the automatization of global intracellular calcium analysis at the single-cell level within a large population simultaneously remains challenging. One software, *CalQuo* (Calcium Quantification), offers some automatic features in calcium analysis. Here, we present an advanced version of the software package: *CalQuo* ^*2*^. *CalQuo* ^*2*^ analyzes the calcium response in the Fourier-domain, allowing the number of user-defined filtering parameters to be reduced to one and a greater diversity of calcium responses to be recognized, compared to *CalQuo* that directly interprets the calcium intensity signal. *CalQuo* ^*2*^ differentiates cells that release a single calcium response and those that release oscillatory calcium fluxes. We have demonstrated the use of *CalQuo* ^*2*^ by measuring the calcium response in genetically modified Jurkat T-cells under varying ligand conditions, in which we show that peptide:MHCs and anti-CD3 antibodies trigger a fraction of T cells to release oscillatory calcium fluxes that increase with increasing k_off_ rates. These results show that *CalQuo* ^*2*^ is a robust and user-friendly tool for characterizing global, single cell calcium responses.

## Introduction

Calcium (Ca^2+^) signaling is a dynamic process that influences a broad range of cellular life^1, 2^. In cell-mediated immunity, Ca^2+^ signaling is crucial for T-cell development, tolerance, and homeostasis^3^. In particular, Ca^2+^ signaling plays a key role in T-cell activation^4, 5^, in which 75% of genes derived from the cDNA of stimulated T cells were found to be Ca^2+^ influx dependent^6^. Specifically, triggering of the T-cell receptor (TCR) following engagement with peptide:MHC (pMHC) leads to the release of Ca^2+^ from the endoplasmic reticulum (ER) by the opening of the inositol 1,4,5-triphosphate receptor (IP_3_R), an ER transmembrane Ca^2+^ channel^7, 8^. Subsequent Ca^2+^ fluxes result from store operated channels (SOC), known as Ca^2+^ release-activation Ca^2+^ (CRAC) channels^7–9^. Various Ca^2+^ responses have been found in cells with correlatively different downstream effects on the activity of transcription factors, e.g. Nuclear Factor of Activated T cell (NFAT), and Nuclear Factor (NF)-κB^10, 11^.

Considering the importance of Ca^2+^ dynamics during T-cell activation, a standardized, automatized method is required in order to effectively study and compare Ca^2+^ responses, especially given their rapid, transient nature and cell to cell variability. When studying Ca^2+^ responses, two key aspects should be taken into account: the method of capturing the Ca^2+^ responses and the subsequent analysis of the response of each cell. Typical methods of capturing Ca^2+^ responses include flow cytometry, plate readers, or fluorescence microscopy^12–14^. The use of flow cytometry and plate readers are limited in scope as they do not provide information of Ca^2+^ responses over time or in individual cells, respectively^12, 14^. Commonly used live-cell fluorescence microscopy can measure Ca^2+^ over time, but the use of fluorescence dyes that change emission upon Ca^2+^-binding typically measure only a small number of cells per experiment and with a poor signal to noise ratio^15, 16^. Automatized analysis methods have been used to quantify Ca^2+^ responses derived from fluorescence microscopy, but they measure Ca^2+^ in localized spikes or microdomains within cells, such as in muscle cells and neurons^17–19^. Few automatized analyses of Ca^2+^ responses quantify global Ca^2+^ responses in non-excitable cells, such as T cells^20, 21^. Ca^2+^ responses are here defined as changes in the global intracellular Ca^2+^ levels on the sub-second to second time-scale. Additionally, those methods that do quantify global Ca^2+^ responses do not automatize the segmentation and detection of cells and the Ca^2+^ based fluorescence intensity (now referred to as Ca^2+^ intensity) within each cell needed to then quantify the Ca^2+^ response^22^.

*CalQuo* is a MATLAB-based software that attempts to overcome these limitations by automatically reading in and segmenting images, allowing for the classification of the global Ca^2+^ intensity of a few thousand cells simultaneously over time and the subsequent quantification of cells with a particular type of calcium response^20^. *CalQuo* analyzes the changes in Ca^2+^ intensity of each cell over time to determine whether a cell has triggered, allowing the fraction of cells that have triggered in the sample population to be quantified^20^. *CalQuo* is compatible with images acquired using a fluorescence spinning-disk confocal microscope, which can image Ca^2+^ responses on the sub-second time-scale^20^. In order to use *CalQuo*, however, several parameters regarding the curve of the Ca^2+^ intensity must be manually input to achieve successful filtering of the data^20^. User-dependent parameters describing the landing event where the cell first makes contact with the activating surface, the peak fluorescent intensity, the time between landing and peak events, and the time-scale of the signal decay are required to characterize a typical curve. Despite the success of this approach, input of these parameters limits the flexibility in which a triggering cell may be recognized and does not differentiate between the various Ca^2+^ responses a cell may have^20^. In addition, determining each parameter is time-consuming and opens the possibility of user error and bias.

**Figure 1:**
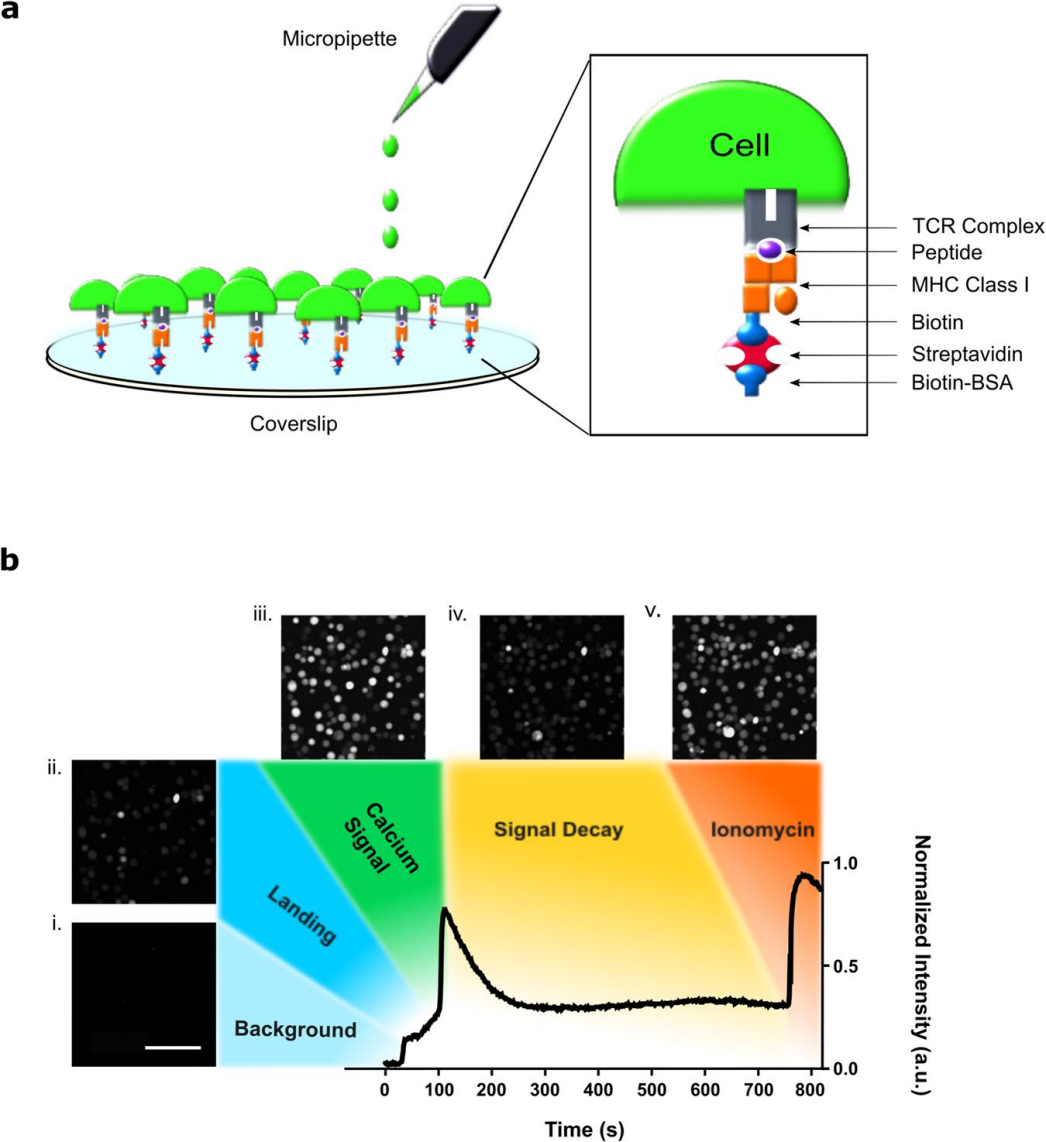
Model of T cell response on antigen functionalized coverslips. (**a**) Diagram of antigen functionalization onto glass coverslips with pMHCs. (**b**) Representative normalized Ca^2+^ based fluorescence intensity plot of a Jurkat T-cell upon landing on a 4D:MHC I functionalized coverslip with a subset of cells from one movie shown in the images taken at (i) 0 s, (ii) 38 s, (iii) 117 s, (iv) 514 s, (v) 764 s. Each circular white circular dot represents 1 cell. Scale bar is 100 µm.

**Figure 2:**
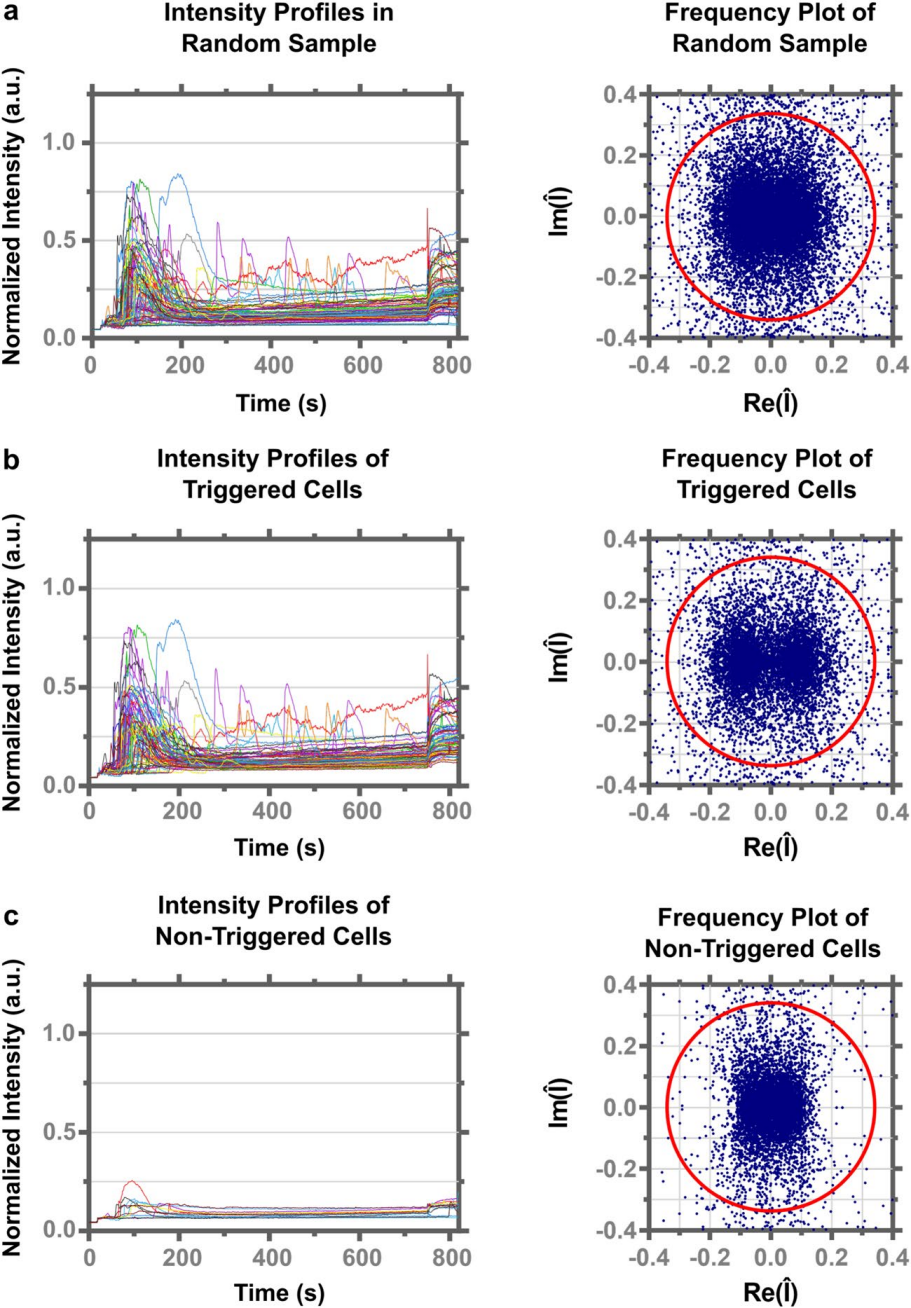
Example plots of normalized Ca^2+^ intensities over time (left) and Fourier transformed Ca^2+^ responses (right) in 1G4-TCR T cells that came into contact with a 9V:MHC functionalized coverslip. (**a**) Representative random sample of the triggering and non-triggering cells. (**b**) Cells of the random sample that *CalQuo* ^*2*^ regards as triggering. (**c**) Cells of the random sample that *CalQuo* ^*2*^ regards as non-triggering. The Triggering radius of 0.34 is depicted as the red circle in the frequency plots.

To address these concerns, we developed an updated version of *CalQuo*, called *CalQuo* ^*2*^, where new algorithms to analyze and characterize Ca^2+^ responses have been developed and implemented. Using antigen functionalized coverslips to trigger T cells and, thus, induce Ca^2+^ release (Fig. 1a), time-course images were taken to visualize the Ca^2+^ intensity response curve of each cell (Fig. 1b). As described by Fritzsche *et al*., the Ca^2+^ based fluorescence intensity over time can be extracted by applying feature recognition to locate each cell followed by the application of distance regularized level set (DRLS) evolution to segment each individual cell and integrate its Ca^2+^ intensity over time. Building upon *CalQuo, CalQuo* ^*2*^ implements the Bio-Formats 5.3.3 toolbox (http://www.openmicroscopy.org/site/products/bio-formats) to accelerate the rate at which an image stack is read into MATLAB (see Materials and Methods). Once the Ca^2+^ response curve has been extracted a Fourier transform of the Ca^2+^ based intensity signal is calculated, allowing filtering of the signal to be carried out based on the phase and frequency of the Ca^2+^ response (see Supplementary Information). Critically, triggering and non-triggering cells have distinct signatures in Fourier space, and choosing a radius in the imaginary (|Im|) or real (|Re|) part on the complex plane allows the two signatures to be separated (Fig. 2). A Fourier transform converts the intensity signal into the dominant components of frequency (real) and phase (imaginary) on the complex plane, thus responses lacking any frequency above the noise will show distributions of the real component accumulated around zero. Conversely, cells that show a distinct frequency will have a distribution away from zero. Because the Fourier transform is also sensitive to the phase of the response, the components on the complex plane can show a broad distribution on the imaginary axis due to the lack of temporal coherence in the responses, which is particularly prominent in non-triggering cells. Empirically, cells lacking a strong Ca^2+^ flux, usually less than 0.2 arbitrary unites (a.u.) above baseline in a normalized intensity curve, will hence have near zero values for their imaginary and real components (Fig. 2c). Likewise, high Ca^2+^ fluxes will result in imaginary and real components of higher magnitudes (Fig. 2b). Thus, a radius, defined here as the triggering radius, around the origin can be determined, and if more than 10% of the data points on the complex plane are outside of the triggering radius, *CalQuo* ^*2*^ considers the cell to be triggering. The triggering radius is the only user determined parameter, significantly reducing the required degrees of freedom. The possibility of automatically determining the triggering radius is also available (see Supplementary Information).

**Figure 3:**
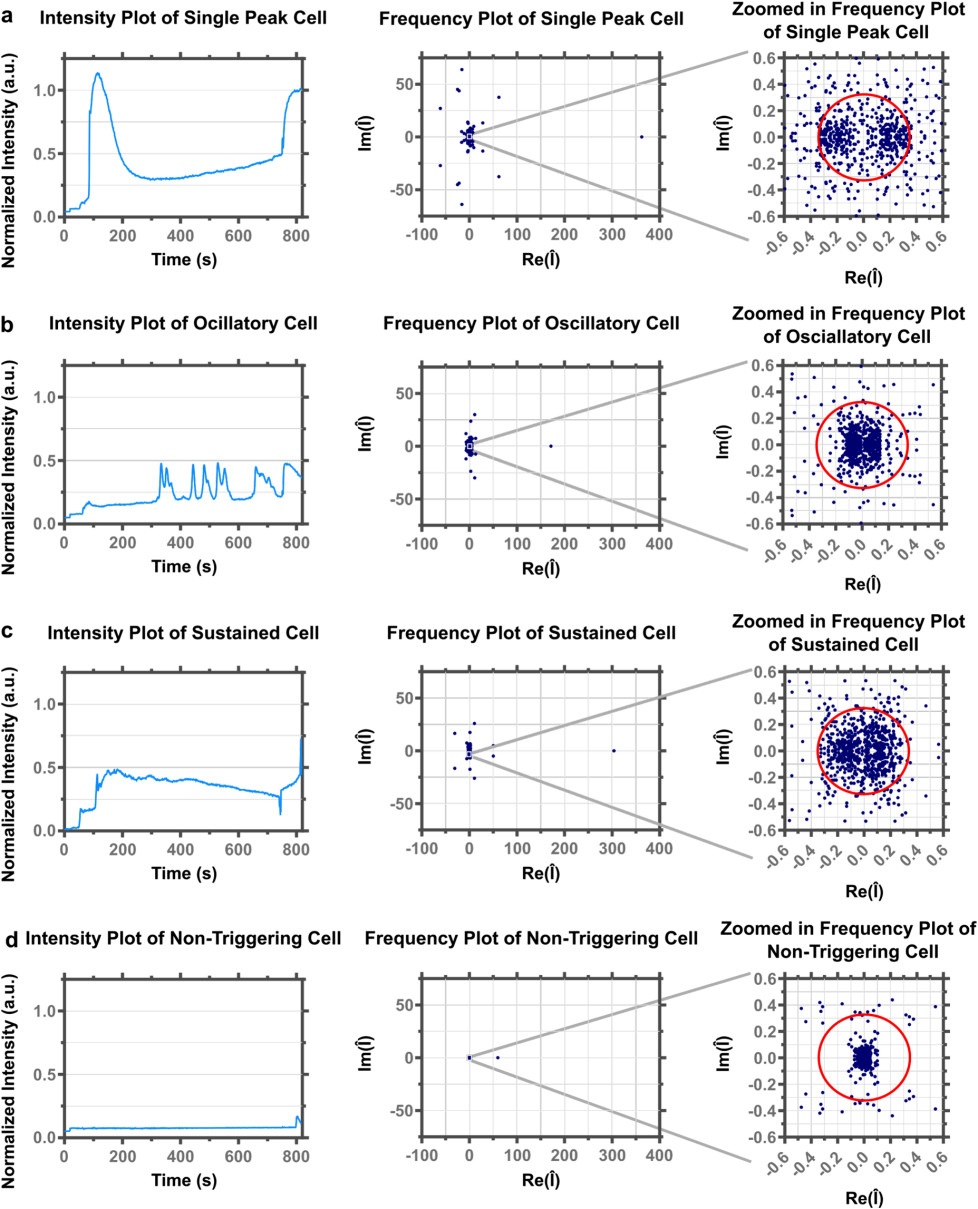
Example plots of normalized Ca^2+^ intensities over time (left) and calcium responses Fourier transformed (middle and right) in individual 1G4-TCR T cells that came into contact with a 9V:MHC functionalized coverslips. The right plots are zoomed in versions of the middle plots. (**a**) A T cell with a single Ca^2+^ intensity peak. (**b**) A T cell with multiple Ca^2+^ intensity peaks (oscillatory). (**c**) A T cell with a sustained Ca^2+^ intensity maximum. (**d**) A T cell without a Ca^2+^ intensity maximum that surpasses 0.2 a.u. The triggering radius of 0.34 is shown as the red circle.

Since the type of Ca^2+^ pattern elicited from a triggering T cell is important in determining the nuclear localization of the transcription factors and thus the type of T cell response^2, 10, 11^, *CalQuo* ^*2*^ further builds upon the capabilities of *CalQuo* by separating cells with a single Ca^2+^ flux from cells with oscillatory Ca^2+^ fluxes. By setting a minimum threshold, defining a peaked response in the derivative of the intensity curve, the program can separate between a cell that displays only a single peak and those that display multiple intensity peaks above the minimum threshold. Cells that show two or more peaks above the minimum threshold are defined as oscillatory. The difference between singular peak, oscillatory, sustained, and no Ca^2+^ flux can be seen in the normalized intensity plots and Fourier domain plots (Fig. 3). The distribution of the components in the Fourier domain differs between the various responses, with single, oscillatory and sustained responses showing higher magnitude components less tightly clustered around zero compared to non-triggering cells. In the following, we refer to singular Ca^2+^ fluxes when the responses show one characteristic peak and to oscillatory responses when multiple peaks are shown over time. We call Ca^2+^ fluxes sustained when they display shallow or no fluorescence decay and no Ca^2+^ flux when any type of response is absent.

In the following, we first validate the performance of *CalQuo* ^*2*^ in distinguishing triggering cells and their Ca^2+^ patterns compared to manual classifications. Following this, we further validate *CalQuo* ^*2*^ by systematically analyzing the Ca^2+^ responses of Jurkat T-cells stimulated by antigens of differing k_off_ rates. Specifically, we find that Jurkat T-cells that were placed on nonspecific glass coverslips had more oscillatory Ca^2+^ fluxes with a longer time period between cell landing and a Ca^2+^ flux in comparison to that of Jurkat T-cells placed on antigen functionalized glass coverslips, which had a higher fraction of cells displaying a single Ca^2+^ flux and a shorter time for Ca^2+^ to flux. Additionally, we found that *CalQuo* ^*2*^ was able to show that a greater fraction of T cells stimulated by pMHCs had single Ca^2+^ fluxes compared to T cells stimulated by anti-CD3 antibodies (Abs), suggesting that oscillatory Ca^2+^ flux responses specify weak stimulation, whereas singular responses signify fully activating T cells. We find that *CalQuo* ^*2*^ was able to successfully demonstrate that pMHCs and anti-CD3 Abs trigger a fraction of T cells to release oscillatory Ca^2+^ fluxes that increase with increasing k_off_ rates.

## Methods

### Cell Line

Wild-type Jurkat E61_57 T-cells were transduced to express 1G4-TCRs and CD8 coreceptors to produce 1G4-TCR Jurkat T-cells, given by Veronica Chang, a former member of the Simon J. Davis laboratory (University of Oxford, UK). The Jurkat clones given by Takamasa Ueno (University of Kumamoto, Japan), were devoid of the TCR α chain, low in the TCR β chain expression, and low in CD4 expression. To transduce these Jurkats, cDNA of the 1G4-TCR’s α and β chains, given by Vincenzo Cerundolo (University of Oxford, UK), were amplified via polymerase chain reaction (PCR). Using the AgeI and KpnI restriction sites, the amplified PCR products were ligated into the region following the gene that encodes the chicken RPTPσ signal peptide of the pHLsec vector, according to Li *et al*.^1^. The inserted sequence was then sub-cloned into the pHR vector, according to the protocol by Aleksic *et al*.^2^. By over-expressing the 1G4 β chain, the original β chains in the E61_57 cells were removed. Using UCHT1, the transfected cells were stained and sorted to keep TCR negative cells. The same process was repeated twice before a final cell line, E61_57_1G4-β^+^ cell line, was produced. The 1G4-α chains were then added to achieve the E61_57_1G4-TCR cell line, which we refer to as the 1G4-TCR Jurkat T cells.

### Cell Culture

1G4-TCR Jurkat T-cells were incubated in a T-75 flask at 37 °C in 5% CO_2_. Cells were maintained at ~5–10 × 10^5^ cells/mL in 25 mL of RPMI media (Sigma-Aldrich, UK). The media was supplemented with 1% of 10,000 units of penicillin and 10 mg/mL of streptomycin (Sigma-Aldrich, UK), 1% of 200 mM L-glutamine (Sigma-Aldrich, UK), 1% of 100 mM sodium pyruvate (Gibco, UK), 1% of 1 M of HEPES (Sigma-Aldrich, UK), and 10% of fetal bovine serum (FBS) (Gibco, UK).

### Sample preparation

2 mL of the 1G4-TCR Jurkat T-cells, with a cell count of 5–10 × 10^5^ cells/mL and a cell viability of 80%, were taken from a T-75 flask and centrifuged at 1500 rpm for 3 minutes to form a pellet. After discarding the supernatant, cells were resuspended in 50 µL of L15 medium (Gibco, UK), 50 µL of HBS (HEPES-buffered saline) buffer containing 2.5 µM of probenecid (Sigma Aldrich, UK), and 1 µL of 1 mM Fluo-4 AM (Invitrogen, UK). Cells were incubated for 5 minutes at 37 °C followed by washing twice in HBS buffer. The HBS buffer contained 150 mM of NaCl and 10 mM of HEPES in distilled water with a final pH of 7.05 and was then supplemented with probenecid to contain 2.5 µM of probenecid in HBS.

### Functionalization of coverslips

All experiments were conducted on 18 mm # 1.5 circular, glass coverslips (Scientific Laboratory Supplies, UK). Each coverslip was first coated with 20 µL of a biotin-BSA:BSA mix, containing biotinylated BSA at a concentration of 200 µg/ml and BSA at a concentration of 800 µg/ml in distilled water. After 1.5 hrs. of incubation with the biotin-BSA:BSA at room temperature, the coverslips were washed three times in 2 mL of PBS. Coverslips were then coated with 20 µL of streptavidin at a concentration of 10 µg/ml and incubated at room temperature for 1.5 hrs. Coverslips were washed three times with 2 mL of PBS. Coverslips were then coated with 20 µL of either biotinylated pMHCs, biotinylated OKT3, biotinylated UCHT1 at a concentration of 10 µg/ml. For negative control coverslips, the addition of antigen at the final step was omitted. After a further 1.5 hrs. of incubating at room temperature the coverslips were washed with 2 mL of PBS three times and stored at room temperature until required for imaging. Coverslips were prepared fresh on the day of the experiment.

### Peptides

The 1G4-TCR binds specifically to the NY-ESO-1_157–165_ peptide^23^. Two variants of the NY-ESO-1_157–165_ peptide were presented on the MHC class I complexes used: one with a mutation to valine at the 165 position (referred now on as 9V) and one with a mutation to aspartate at the 160 position (referred now on as 4D)^23^. Peptides were synthesized by Generon (UK).

### Biotinylation of Protein

Protein biotinylation was required to specifically functionalize glass surfaces. To achieve biotinylation, an N-Hydroxysuccinimidet (NHS)-ester labelling method was used that targets amine groups typically found on free lysines within the protein. All proteins to be biotinylated were first passed through a desalting column (7 K Zeba Spin, ThermoFisher, UK) to remove any buffer components that may interfere with the biotinylation reaction (e.g. Tris). The reaction was conducted in a volume of 200 μL and a protein concentration of 1 mg/ml. The pH of the solution was adjusted to pH 8 using a 1 M solution of NaOH. Biotin-NHS (EZ-Link, ThermoFisher) was added to the protein solution at a molar ratio of 10:1 and incubated for 1 h at 37 °C. Following incubation, the solution was passed through a further desalting column to remove any unbound biotin.

### Imaging

A Zeiss spinning disk confocal microscope equipped with a 10x air 0.45 NA objective and a pinhole diameter of 50 µm was used to image the Ca^2+^ flux. All imaging was conducted at 37 °C and at 5% CO_2_. The Ca^2+^ flux was visualized by the excitation of the calcium sensitive dye Fluo-4 AM with 488 nm laser light set to 70% of the maximum output power (measure at the objective to be 0.52 mW). The movies were captured using an AxioCam MRm camera. Each experimental acquisition was 1000 frames long, with a frame time of 820 milliseconds, set to maximize signal to noise whilst capturing sufficient temporal resolution. Ionomycin was added during the final 100 frames (82 s). Frame rates and laser powers were chosen to reduce photo-bleaching while maintaining sufficient temporal resolution to capture the dynamics of the Ca^2+^ response. By integrating the intensity of the whole acquisition frame over time, it is possible to appreciate how on the time scale of the Ca^2+^ response, the level of photo-bleaching is minimal (Fig. S2).

For each acquisition, a functionalized coverslip was placed into a circular holder and then covered with 600 µL of HBS buffer. In all cases, the coverslip was given sufficient time to equilibrate to the environmental conditions on the microscope. This ensured that sample focus drift due to temperature fluctuations was minimal during the acquisition. For each acquisition, the correct focal plane was selected by depositing a sparse distribution of labeled cells. Following this, the acquisition was initiated and 50 uL of labeled cells pipetted onto the coverslip, allowing the first contact of the cells and the coverslip to be captured across a large field of view (250 × 250 µm).

## Results

Previous work has shown that the kinetic parameters of the antigen result in differing Ca^2+^ responses in Jurkat T-cells^24^ but manual analysis makes large scale quantification of these differing and complex responses challenging. We exemplified the use of *CalQuo* ^*2*^ by analyzing the Ca^2+^ patterns in Jurkat T-cells interacting with glass coverslips functionalized with antigens of differing binding affinities (Fig. 1a, Supplementary Movies S1–S5). To this end, Jurkat T-cells were transduced to express the specific 1G4-TCR and the co-receptor CD8, allowing stimulation via two variants of NY-ESO-1_157–165_ peptides referred to as 9V and 4D, when presented on the MHC class I molecule^24, 25^. Since anti-CD3 Abs are commonly used to stimulate T cell triggering/activation *in vitro* ^14, 26, 27^, UCHT1 and OKT3 were used in addition to the pMHCs. Specifically, the range of antigens differ in their binding kinetics, with UCHT1 having the lowest dissociation rate k_off_, followed by 9V:MHC, OKT3 and 4D:MHC (Supplementary Information, Table S1). The dissociation rate, k_off_, is inversely proportional to the time period, t_off = _1/k_off_, the TCR stays bound to the ligands.

The Ca^2+^ levels of the Jurkat T-cells were measured using Fluo-4 AM, an acetoxymethyl (AM) esterified Fluo-4 that allows the dye to permeate the cell membrane, upon which a de-esterified Fluo-4 acts as a fluorescent indicator with a 100-fold increase in fluorescence intensity when bound to Ca^2+^ ^28^. A Zeiss spinning disk confocal scanning microscope was used to visualize the Ca^2+^ response with a numerical aperture (NA) of 0.45 and a frame rate of 1.2 frames per second (s). After 900 frames (738 s), ionomycin was added to ensure that each cell could release Ca^2+^ from its ER stores. Furthermore, the cell(s) showing the greatest fluorescence intensity on addition of ionomycin was used to normalize the fluorescence intensity across the population of cells to account for possible inhomogeneous loading of Fluo-4 AM in each acquisition (Fig. 1b).

**Figure 4:**
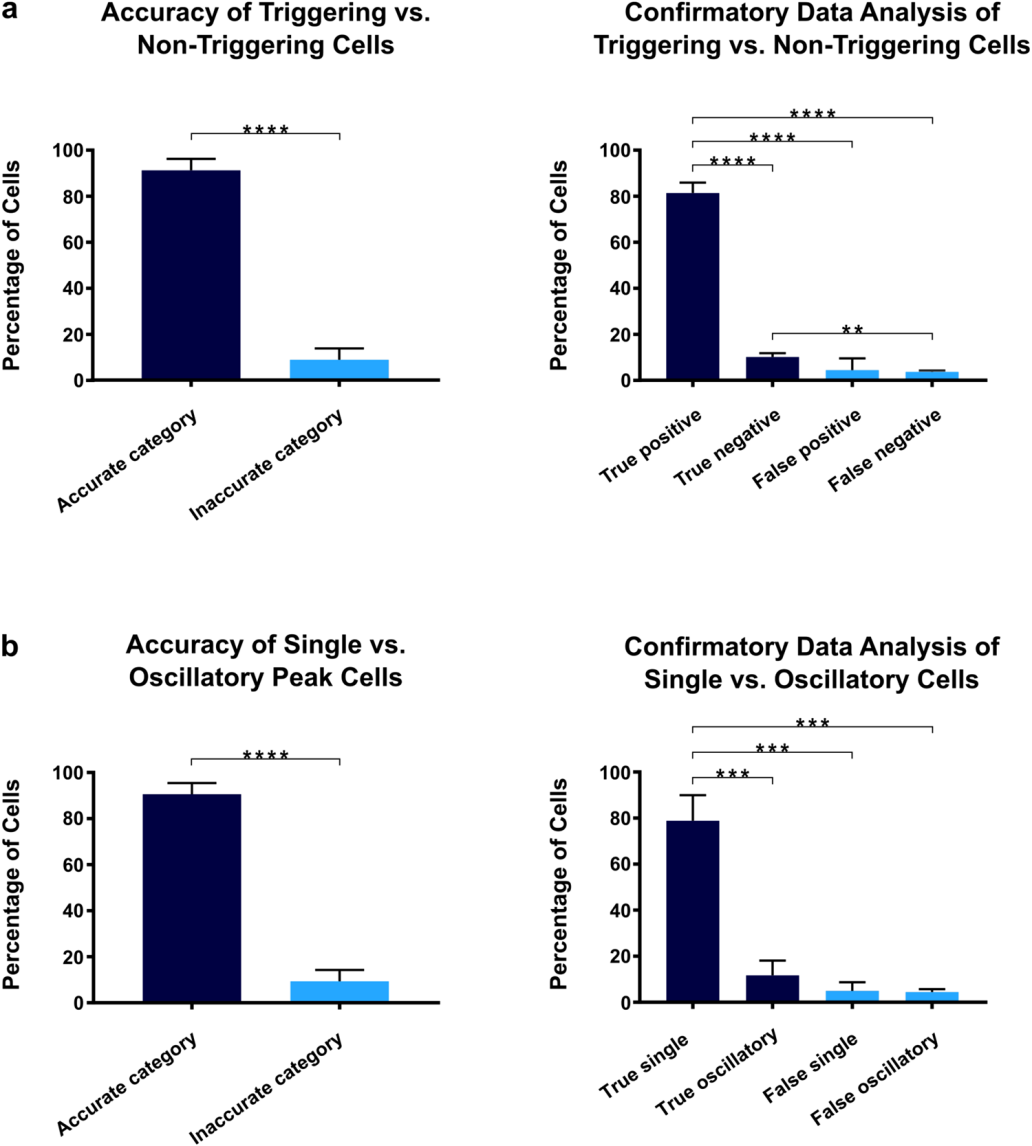
*CalQuo* ^*2*^ compared to manual classifications of Ca^2+^ responses. Comparison of *CalQuo* ^*2*^ versus manual classification in 100 randomly selected cells in three replicates each of T cells that had landed on 9V:MHC functionalized coverslips. *Indicates p ≤ 0.05, **indicates p ≤ 0.01, ***indicates p ≤ 0.001, ****indicates p ≤ 0.0001. (**a**) Comparison between *CalQuo* ^*2*^ and manual classifications of triggering and non-triggering cells. (**b**) Comparison between *CalQuo* ^*2*^ and manual classifications of single and oscillatory Ca^2+^ peak cells. The figures on the left depict the percentage of cells whose Ca^2+^ response *CalQuo* ^*2*^ categorized in a similar manner to the manual categorization with the manual categorization considered to be the accurate depiction of the Ca^2+^ response. The figures on the right further detail the percentage of cells that were accurately classified, as well as the percentage of type I (false positive) and type II errors (false negative).

To first characterize the accuracy of *CalQuo* ^*2*^ in determining triggering and non-triggering cells, we compared the manual classification of 100 random cells over three experimental replicates to the performance of *CalQuo* ^*2*^. In a random sample of 100 cells selected from﻿ each of the 3 replicates﻿ in which T cells had landed on the 9V:MHC functionalized coverslips, *CalQuo* ^*2*^ accurately identified a triggering verses non-triggering Ca^2+^ response on average of 91 ± 4% of the time (Fig. 4a). The difference between accurate and inaccurate characterizations of the Ca^2+^ responses were significant with p < 0.0001. No significant difference was found between the false positives and false negatives, indicating that *CalQuo* ^*2*^ had not biased the data toward either triggering or non-triggering responses (see Supplementary Information).

To further asses the performance of *CalQuo* ^*2*^, we quantified its ability to distinguish single and oscillating responses. Compared to visual inspection and manual sorting of Ca^2+^ responses, *CalQuo* ^2^ accurately separated oscillatory and single peak Ca^2+^ responses on average of 90 ± 4% of the time in the 100 cells picked in a random sample in each of the three replicates in which the T cells had landed on the 9V:MHC functionalized coverslips (Fig. 4b). The difference between accurate and inaccurate characterizations of the Ca^2+^ responses were significant with p < 0.01. No significant difference was found between the percentage of false single and false oscillatory (see Supplementary Information).

In addition to Ca^2+^ response classification, *CalQuo* ^*2*^ is able to quantify the time between the landing of the cell and increase in Ca^2+^ intensity which is referred to as the triggering time. Using the derivative of the Ca^2+^ intensity to find peaks, using the MATLAB ‘findpeaks’ function (https://uk.mathworks.com/help/signal/ref/findpeaks.html), the triggering time was determined by subtracting the time of the lowest peak from the highest peak. The triggering time, period of time that passed between cell landing and the first flux of Ca^2+^; triggering fraction, the fraction of cells that triggered; single peak fraction, fraction of triggered cells with only one Ca^2+^ influx; and oscillatory peak fraction, fraction of triggered cells with multiple Ca^2+^ influxes, were measured to characterize the Ca^2+^ response for each antigen coated surface (see Supplementary Information).

**Figure 5:**
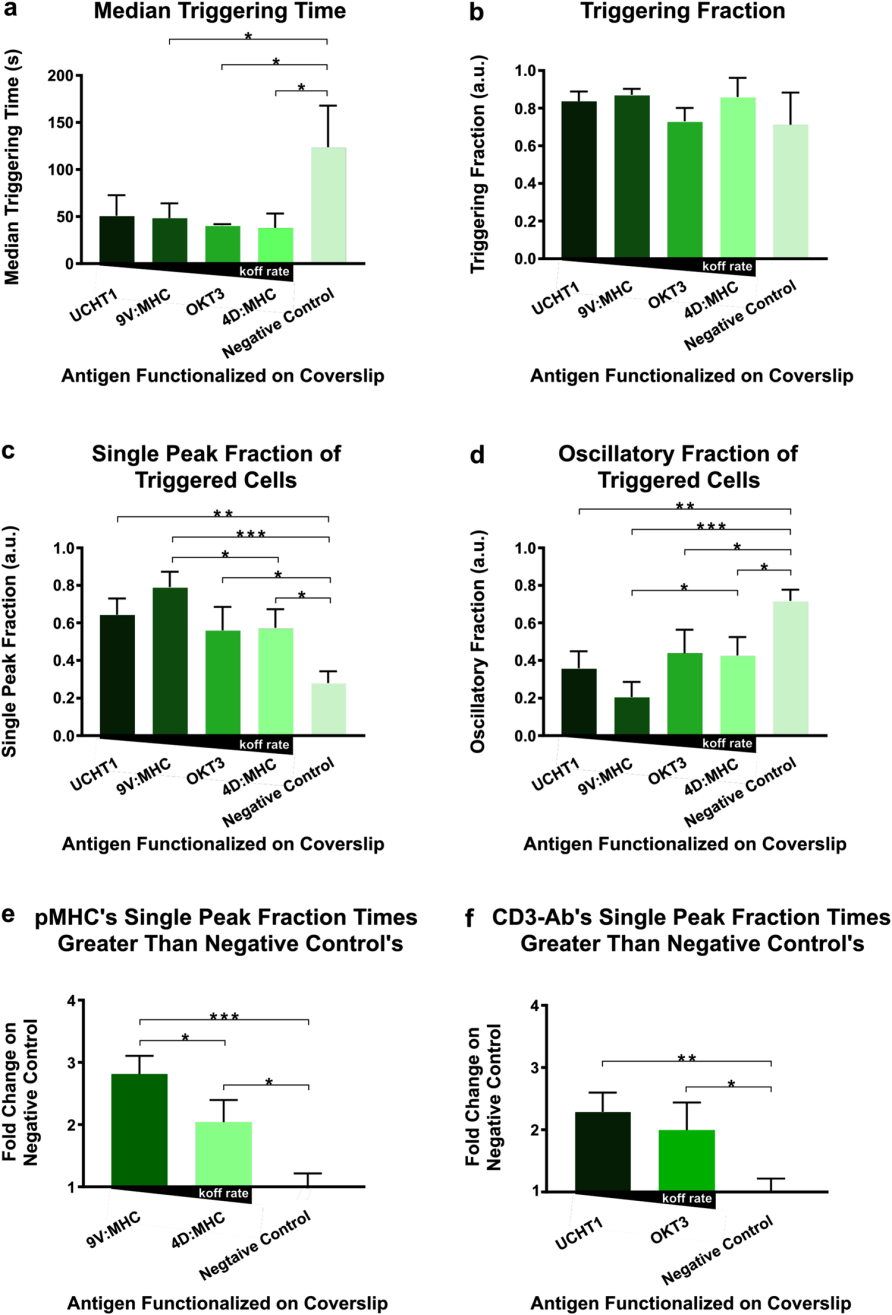
Ca^2+^ patterns in T cells when interacting with pMHC or CD3-antibody functionalized coverslips of varying k_off_ rates. The antigen functionalized coverslips are in order of increasing k_off_ rates from left to right of each graph. Each graph, except the Median Triggering Time, depicts the mean of the replicates with the standard deviation shown with error bars. N = 400 to 1500. *Indicates p ≤ 0.05, **indicates p ≤ 0.01, ***indicates p ≤ 0.001, ****indicates p ≤ 0.0001. (**a**) The Median Triggering Time, the time between landing and the first Ca^2+^ flux. (**b**) The Triggering Fraction. (**c**) The triggered T cells’ Single Peak Fraction. (**d**) The triggered T cells’ Oscillatory Fraction. (**e**) The Single Peak Fraction fold change of the T cells triggered by pMHC on those by the (antigen free) negative coverslip. (**f**) The Single Peak Fraction fold change of the T cells triggered by anti-CD3 Ab on those by the (antigen free) negative coverslip.

Next, to test the ability of *CalQuo* ^*2*^ to quantify differences in the Ca^2+^ response to differing peptides, Jurkat T-cell were systematically exposed to different pMHCs and their Ca^2+^ responses quantified and classified by *CalQuo* ^*2*^ (Supplementary Movies S1–S5). By comparing the mean of the three replicates’ median triggering time, the T cells interacting with the negative control coverslip had a higher mean of median triggering time than that of cells that landed on antigen functionalized coverslips (Fig. 5a). A significant difference was found between the mean of median triggering time of the cells that landed on the negative control coverslip and of the cells that landed on 9V:MHC, 4D:MHC, and the OKT3 functionalized coverslips, with p = 0.0493, 0.0336, and 0.0311, respectively. The difference between cells that landed on UCHT1 and the negative control coverslip missed significance with p = 0.0623 (see Materials and Methods). There was no significant difference found among the mean of median triggering time of cells that landed on antigen functionalized coverslips. To analyze the triggering time, the median was used instead of the mean because the distribution of the triggering time of the cells in each replicate displayed a long tail.

To further test the ability of *CalQuo* ^2^ to quantify Ca^2+^ response differences to varying antigens, especially its ability to separate single and oscillatory calcium fluxes, the triggering fraction, single peak fraction, and oscillatory peak fraction were analyzed. Because these quantitative fractions were normally distributed, the arithmetic mean and standard deviation was used. Unexpectedly, *CalQuo* ^2^ was able to show that there was no significant difference found between the mean triggering fraction of the cells that landed on the negative control coverslips and that of the cells that landed on the functionalized coverslips (Fig. 5b). Consistently, no significant difference was found among the mean triggering fraction of the cells landing on the four different functionalized coverslips (Fig. 5b). However, when analyzing the single peak fraction of cells that triggered, the mean of the replicates’ single peak fraction of cells that landed on functionalized coverslip were significantly higher than that of cells landed on the negative control coverslip (Fig. 5c). Comparing the computed mean single peak fraction of triggered cells placed on the negative control coverslip and that of triggered cells placed on antigen functionalized coverslips in order of increasing k_off_ rates, the p-value was significantly different in all three cases (p < 0.01). Additionally, the mean single peak fraction of the triggered cells that landed on the 9V:MHC coverslip was significantly greater than that of triggered cells that landed on the 4D:MHC coverslip by 0.217 a.u. with p = 0.039. The reverse was seen in the oscillatory peak fraction of triggered cells (Fig. 5d). Addition of the calcium chelator, ethylene glycol-bis(β-aminoethyl ether)-N,N,N′,N′-tetraacetic acid (EGTA), to investigate the effects of residual calcium levels remaining in the media on oscillatory Ca^2+^ responses, showed that the fraction of triggering cells decreased, suggesting that calcium present in the imaging medium was contributing to the fraction of oscillatory responses observed on negative control and weakly activating coverslips (see Supplementary Information Fig. S1).

This *CalQuo* ^2^ analysis revealed an overall monotonic trend that could not be seen between the mean of the replicates’ single peak fraction of the triggered cells and their k_off_ rates, but a monotonic trend could be seen within the type of antigen (pMHC or Ab). For example, despite UCHT1 having the lowest k_off_ rate among the four antigens used, T cells exposed to 9V:MHC had the highest single peak fraction, which was significantly higher than T cells exposed to 4D:MHC. Additionally, despite OKT3 having a similar k_off_ rate to 9V:MHC, which are 0.39 s^−1^ and 0.33 s^−1^, respectively^23, 29, 30^, T cells contacting OKT3 did not have a significantly higher single peak fraction than 4D:MHC, which has a k_off_ rate of 2.59 s^−1 23^. However, when evaluating cells stimulated by only pMHCs or by only Abs, the mean single peak fraction of triggered cells monotonically decreases with increasing k_off_ rates, especially in regards to cells stimulated by pMHCs (Fig. 5e and f). Cells triggered both by either 9V or 4D had significantly higher mean single peak fractions than cells landing upon the negative control, and cells triggered by 9V had a significantly higher mean single peak fraction than that of cells exposed to 4D (Fig. 5e).

## Discussion

From the raw fluorescent images, *CalQuo* ^*2*^ is able to segment image stacks and automatically characterize and filter the resulting Ca^2+^ response curves. *CalQuo* ^*2*^ accelerates the image read-in speed into MATLAB, using the Bio-Formats toolbox, and detects individual cells and tracks their Ca^2+^-based fluorescence intensity over time using feature recognition algorithms. Using Fourier space analysis, the software is able to determine which cells have a Ca^2+^ flux above the noise profile and using the minimum threshold peak, separates cells into those with single Ca^2+^ fluxes or with multiple Ca^2+^ fluxes. After characterizing Ca^2+^ responses in individual cells, *CalQuo* ^*2*^ characterizes the Ca^2+^ response of the whole sample population by determining the described parameters. By subtracting the time frame of the highest peak and second highest peak, found using the derivative of the Ca^2+^ intensity, *CalQuo* ^*2*^ determines the triggering time of each cell. Additionally, the Ca^2+^ intensity amplitude and Ca^2+^ intensity peak width is also provided by *CalQuo* ^*2*^ (see Supplementary Information). By analyzing the response function in Fourier space, *CalQuo* ^*2*^ is robust against noise in the raw data, offering improved performance over algorithms based on a single threshold^21^.

Comparing the quantifications of Ca^2+^ responses both manually and by *CalQuo* ^*2*^, we demonstrate the capacity of *CalQuo* ^*2*^ to differentiate between triggering and non-triggering cells and between single and oscillatory cells without biasing the data towards one category over another. Thus, *CalQuo* ^*2*^ successfully employs the Fourier transform and just one user-defined parameter, the triggering radius, to categorize calcium responses as either triggering or non-triggering. Analyzing calcium responses transformed into the Fourier space removes the need to specify each of the five parameters defining a single, specified model of a calcium curve, as was required for the previous version of *CalQuo*. Reduction in these user input parameters increases the flexibility in which a triggering calcium curve can be identified and lowers the introduction of user bias and error. Moreover, users with limited programming and computational ability can more easily use and access this quantitative Ca^2+^ analysis tool, broadening the application of such a software. On top of enhanced performance, *CalQuo* ^*2*^ offers the ability to separate cells with single and oscillatory calcium responses.

Using *CalQuo* ^*2*^ to analyze the triggering time, we were able to show that T cells interacting with uncoated negative control coverslips took longer to release Ca^2+^ than T cells interacting with antigen functionalized coverslips. The triggering fraction, however, did not show a significant difference in the Ca^2+^ response between T cells landing on antigen functionalized and on non-antigen functionalized glass coverslips. Nonetheless, when separating the cells into those that express a single Ca^2+^ flux and those that express oscillatory Ca^2+^ fluxes, statistically significant differences were found between the T cells placed on the UCHT1, 9V:MHC, OKT3, or 4D:MHC functionalized coverslip and the T cells placed on the negative control coverslips. And, although a monotonic negatively correlated trend could not be seen between the k_off_ rates and the single peak fraction of the triggered cells, when separating these T cells into those that were stimulated by pMHCs and those by anti-CD3 Ab, the single peak fraction were seen to decrease with increasing k_off_ rates. These results indicate that while the k_off_ rate of an antigen is important in determining the type of Ca^2+^ responses induced in T cell, the type of antigen and where that antigen binds/stimulates the TCR-CD3 complex are also significant factors in the type of Ca^2+^ response.

*CalQuo* ^*2*^ is a software capable of quantifying large populations of cells displaying diverse Ca^2+^ responses. This will be important when links are to be drawn between the nature of the calcium signaling and the wider biological function. Single, oscillatory, and sustained Ca^2+^ fluxes have been observed in various studies^14, 16, 20, 28^. Wülfing *et al*.^21^ propose that the different Ca^2+^ patterns may arise from different transformations of the T cells used, with hybridomas displaying greater oscillatory Ca^2+^ responses. In the presented experiments, one type of T cell is used, and different Ca^2+^ responses are seen under different ligand conditions, suggesting that the strength and type of stimulus affect the pattern of the Ca^2+^ response. A single Ca^2+^ flux is consistent with the type of response seen in wild type Jurkat T - cells^20^, while oscillatory Ca^2+^ fluxes may be indicative of a T cell trying to increase the sensitivity to weaker stimuli, since there is not a linear correlation between Ca^2+^ levels and activation of transcription factors^10, 28^. Therefore, it is believed that single Ca^2+^ flux on a twelve-minute time-scale is indicative of a stronger triggering response than an oscillatory Ca^2+^ response. Without *CalQuo* ^*2*^, we would not be able to demonstrate the differences between single and oscillatory Ca^2+^ responses in a quantitative manner, thereby forfeiting the ability to understand the biological importance of Ca^2+^ in T-cell triggering and activation. Crucially, *CalQuo* ^*2*^ addresses the need within the field, providing a software capable of quantifying diverse Ca^2+^ responses necessary to understand the broader functional role of such responses.

## Conclusion

In conclusion, *CalQuo* ^*2*^ is an improved MATLAB-based version of *CalQuo*, capable of automatizing the analysis of Ca^2+^ responses found in single cells and within the whole sample population. With only one manual parameter that can optionally become automatic, there are less degrees of freedom with *CalQuo* ^*2*^, increasing the accuracy in which cells are considered to be triggering or non-triggering based on their Ca^2+^ response. The automatization of *CalQuo* ^*2*^ without any complex mathematical computation required of the user makes *CalQuo* ^*2*^ easy to use and reduces user bias and error. CalQuo^2^ is suitable for analyzing Ca^2+^ responses in non-excitable cells at sub-second time-scales.

## Electronic supplementary material

Supplementary Information

Supplementary Movie 1

Supplementary Movie 2

Supplementary Movie 3

Supplementary Movie 4

Supplementary Movie 5
