# Supplementary material for: CalQuo2: Automated Fourier-space, population-level quantification of global intracellular calcium responses
Source: Sci Rep. 2017 Jul 14;7:5416. doi: 10.1038/s41598-017-05322-z (PMC5511169; doi:10.1038/s41598-017-05322-z)
Supplement: Supplementary file 1 — Supplementary Information [file 41598_2017_5322_MOESM1_ESM.pdf]

SUPPLEMENTARY INFORMATION

CalQuo²: Automated Fourier-space, population-level quantification of global intracellular calcium responses

Angela M. Lee[1$], Huw Colin-York[1$] , Marco Fritzsche[1,2*]

[1]MRC Human Immunology Unit and [2]Wolfson Imaging Centre, Weatherall Institute of Molecular Medicine, University of Oxford, Headley Way, OX3 9DS Oxford, United Kingdom.

[2]Kennedy Institute for Rheumatology, Roosevelt Drive, University of Oxford, OX3 7FY Oxford, United Kingdom.

*Correspondence to: marco.fritzsche@rdm.ox.ac.uk
$ Authors contributed equally

Table of Contents

I. Analysis of Calcium Data 3
II. CalQuo^2 Software 3
III. CalQuo^2 User Manuel 4
IV. Effects of Residual Ca^{2+} 7
V. Supplementary Table 9
VI. Supplementary Figures 10
VII. Supplementary Movies 12

I. ANALYSIS OF CALCIUM DATA

i. Pre-processing of calcium data. For each calcium response movie, ImageJ was used to convert the original 16-bit, CZI files to 8-bit, TIFF files. In addition, a sliding paraboloid was used to subtract background with a rolling ball radius of 50.0 pixels. Following this preprocessing step, each TIFF stack was loaded into the CalQuo^2 software using the CalQuo20_read.m script. Briefly, CalQuo^2 is able to localize each cell within the image stack and extract the fluorescence intensity of each cell at each time point, producing a calcium response curve for each cell. Response curves are normalized to the brightest cell in the acquisition. A second script, CalQuo20_analysis.m, allows filtering of the response curves and hence quantification of the population of cells, outputting the triggering fraction (the fraction of cells that show an elevated level of calcium on coming into contact with glass surface), triggering time (the average time taken by the population of cells to show an elevated level of calcium), and oscillatory fraction (the fraction of cells that display an oscillatory calcium response). See the following sections for a more detailed discussion of the software.

ii. Statistics. Unpaired t-tests were used to determine the statistical significance of the differences found in the triggering fraction, single peak fraction, oscillatory fraction, and triggering time between the varying antigen stimulated T cells. If a normal distribution among the replicates was seen in a given parameter, the arithmetic mean and standard deviation was found. If the replicates displayed a skewed distribution, the median and standard deviation was found. Typically, the calcium data were normally distributed but otherwise directly indicated in the main manuscript text.

II. CALQUO^2 SOFTWARE

The CalQuo^2 package, written in MATLAB, can be downloaded using the following link - http://www.bpi-oxford.com/software/. The package includes two master files: ‘CalQuo20_read.m’ and ‘CalQuo20_analysis.m.’ The ‘CalQuo20_read.m’ reads 8-bit TIFF-stack images from a user-defined folder, detects cells defined throughout the time-lapsed image, and determines the calcium-based intensity in each frame of each detected cell using a combination of feature detection and DRLS. The TIFF-stacks can be of differing pixel sizes. ‘CalQuo20_analysis.m’ can then analyze the output data of the ‘CalQuo20_masterfile.m’ in the MATLAB workspace. Filtering of the calcium responses is achieved by transforming the intensity trace of each cell into the Fourier domain via MATLAB’s discrete Fourier transform algorithm. Another file, called ‘CalQuo20_parameters.m,’ sets the parameters independently required in the ‘CalQuo20_read.m’ and ‘CalQuo20_analysis.m’ files. Because the master files read in the parameters file independently, the user can optimize the analysis parameters without affecting the raw data processed by the read in file. The outputs of CalQuo^2, specifically of the ‘CalQuo20_analysis.m’ file, are organized in file-structures, found in the MATLAB workspace saved as, RESULTS. The RESULTS file reports information on the triggering, triggering time, calcium profile, and triggering peak, of

both the individual cells and of the overall population. The values corresponding to individual cells can be found in the structure files.

'CalQuo20_read.m' uses the BioFormat 5.3.3 toolbox (www.openmicroscopy.org/site/products/bio-formats) to read images into MATLAB. Note, please download the MATLAB BioFormat 5.3.3 toolbox, separately from *CalQuo²* and add it to the MATLAB path. It then uses the MATLAB feature recognition, called 'findfeature2d' (http://people.umass.edu/kilfoil/downloads.html), and distance regularized level set evolution (DRLS) algorithms to segment each cell from the image stack, allowing accurate quantification of the calcium intensity at each time point.

Once all calcium response curves have been quantified and normalized, the curves can be filtered and classified. By first taking a Fourier transform of each response curve the response can be analyzed in terms of frequency and phase in the complex plane. By defining a so called triggering radius defined on the complex plain, curves showing a response exceeding the triggering radius on the complex plain in defined as triggered.

III. CALQUO² USER MANUAL

To run *CalQuo²*, the path must first be set by selecting 'Set Path' on the MATLAB 'Home' panel. All of the CalQuo² files and sub-files should be under one folder, called *CalQuo²*, therefore, clicking on the 'Add with SubFolders' tab should include all of the files required. If the path is saved, there is no need to set the path in the future. Only three files need to be open in Editor, CalQuo20_read.m,' 'CalQuo20_analysis.m,' and 'CalQuo20_parameters.'

i. Read images and detect cell features

Before running the 'CalQuo20_read.m,' there are twelve parameters that need to be set:

<u>Software control parameters:</u>

**PARAMETERS.frametime**	The frame time at which data was collected by the microscope.
**PARAMETERS.lastframenumber**	The last frame number of the movie (number of all images) or the last frame before addition of ionomycin.

Profile control parameters:

**PARAMETERS.ionorm**	Determines whether to use the ionomycin frame to normalize the intensities. A quantity of *0* turns off the ionomycin frame normalization, in which the brightest detected cell of the last frame will be used to normalize the data. A quantity of *1* turns on the ionomycin frame normalization.
**PARAMETERS.ioframe**	The ionomycin frame number by which the brightest cell is determined for normalization.

Feature recognition parameters

**PARAMETERS.feature_size**	The approximate feature size in pixels.
**PARAMETERS.subregion_size**	Twice the feature size.
**PARAMETERS.feature_threshmin**	The defined percentage of features with the smallest intensities excluded from final cell feature detection.
**PARAMETERS.feature_threshmax**	The reciprocal of the defined percentage of features with the greatest intensities excluded in final cell feature detection.

DRLS parameters

**PARAMETERS.mode**	The mode for detecting cell feature locations. A quantity of *0* uses the feature edge and is recommended for more precise cell locations. A quantity of *1* roughly estimates the cell periphery and is recommended for cells that drift within the course of the experiment.
**PARAMETERS.iter_inner**	The minimal polygon radius size in pixels.
**PARAMETERS.iter_outer**	The maximal polygon radius size in pixels.

The outputs of ‘CalQuo20_read’ include information regarding the cell detection and features, which can be found in the MATLAB Workspace, under RAWDATA.files{1,1}, and one figure that opens as a new window. The figure is an image of the last frame of the image stack, with blue circles representing a cell feature that has been detected by ‘CalQuo20_read’. If there are blue circles that encircle areas of background and not actual cells or if dimmer cells have not been detected, then the **PARAMETERS.feature_threshmin** needs to be adjusted accordingly. If there are very bright, saturated or dead cells, the **PARAMETERS.feature_threshmax** can be adjusted to exclude those cells, up to the discretion of the user. RAWDATA.files{1,1} contains the spatial coordinates and the normalized intensity of each detected cell.

ii. Analyze RAWDATA

After running ‘CalQuo20_read.m’ with acceptable RAWDATA outputs in the MATLAB workspace, it is suggested to save the workspace, in order to maintain the RAWDATA outputs when adjusting the parameters for the analysis. Before running the ‘CalQuo20_analysis,’ there are four parameters that need to be set.

**PARAMETERS.autoindex**	Determines whether to automatically or manually choose the frequency triggering radius. A quantity of *0* turns off the automatized triggering radius selection, while a quantity of *1* turns it on.
**PARAMETERS.Sensitivity**	The step-size in the frequency space for automatized triggering radius computation and selection (not used if **PARAMETERS.autoindex** set to 0)**.**
**PARAMETERS.Trigger_radius**	The manually determined triggering radius in the frequency space. If more than 10% of the complex numbers in the frequency space are outside of the triggering radius, then the cell is considered to be triggering. The origin of the circle is (0, 0) in the complex plain. Cells that are triggering should have at least one normalized intensity peak above the threshold, which is ~0.2 a.u. above baseline signal.
**PARAMETERS.minTreshPeak**	The minimum fluctuation in the intensity signal for the fluctuation to be considered a peak. This parameter is used to determine whether a cell is oscillatory.

It is possible to check if the right parameters for determining whether a cell is triggering have been chosen by viewing the normalized, random sample intensity plots of the 'Trigger Response Function' versus the 'Non-Trigger Response Function'. These two plots are an output of 'CalQuo20_analysis,' and they show a random sample of 10% of the data each. If the right parameters have been set, then almost all of the cells that have an intensity peak ~0.2 a.u. or more above the baseline should be in the 'Trigger Response Function' plot and almost all of the cells that have an intensity peak less than ~0.2 a.u above baseline should be in the 'Non-Trigger Response Function' plot. Alternatively, the 'plot_intensities_triggeredcells.m' function can be opened in Editor and run to view the 'Trigger Response Function' and 'Non-Trigger Response Function' plots of individual cells. If the 'Trigger Response Function' and 'Non-Trigger Response Functions' plots display intensity functions that are in the wrong plot, it is recommended to keep the **PARAMETERS.Sensitivity** constant at 0.001 and adjust the **PARAMETERS.Trigger_radius** at an increment/decrement of 0.1 to start.

In order to check if the right quantity was chosen for the **PARAMETERS.minTreshPeak**, the 'plot_intensities_osc' file can be opened in Editor and run. Cells with two or more intensity peaks ~0.2 a.u. above baseline should have an intensity profile plotted onto the 'Oscillatory Peak' plot, while the intensity profile of cells with only one peak above the threshold should be in the 'Single Peak' plot. Furthermore, cells with no peaks above the threshold should be the 'Non-Trigger' plot.

The outputs of 'CalQuo20_analysis' include statistics and indexes found in the MATLAB Workspace, labeled RESULTS, and two figures that appear in two new windows. One figure displays two plots, 'Trigger Response Function' and the 'Non-Trigger Response Function,' as mentioned above. The other figure displays boxplots of the triggering fraction, triggering time, and triggering radius, as well as a histogram of the triggering time. Under RESULTS.files{1,1}, the population level statistics, including the TotalNumCells (the total number of cells detected), NumTriggeringCells (the total number of cells that triggered), the TriggerFraction (triggering fraction), the PeakOscFraction (oscillatory peak fraction), and the TriggerRadius (triggering radius), are displayed. The average, median, and standard deviation of the TriggerTime (time between landing and the first peak) and the TriggerPeak (normalized intensity of the first peak) are also displayed. Furthermore, individual cell outputs can be found in the indexes within the RESULTS.files{1,1}. Such indexes include the LandingPeak (time that cells landed), TriggerIndex (whether a cell has triggered), TriggerTime, TriggerPeak, TriggerFrame (time when a cell has its first intensity peak), and SinglePeakIndex (whether a cell has an oscillatory intensity).

IV. EFFECTS OF RESIDUAL Ca^{2+}

For all conditions examined in the main text, cells were imaged in a medium containing HBS. Because HBS contains no additional Ca^{2+}, the only source of Ca^{2+} that was present was in the cell itself and any residual Ca^{2+} remaining from the L15 buffer used

when incubating the cell with the Fluo4-AM dye or from the full cell culture medium after washing. To investigate whether the low residual levels of Ca^{2+} remaining in the media during the experiments was contributing to the observations outlined in the main text, a set of experiments was conducted in the presence of the Ca^{2+} chelator, EGTA. In these experiments, cells were washed and suspended in either an HBS buffer only or 1mM of EGTA in the HBS buffer, and 600 µL of the EGTA in HBS buffer was used to cover the coverslip. The results show that in the presence EGTA, the fraction of triggering cells, particularly those that oscillate, decreases, suggesting that Ca^{2+} present in the imaging medium was contributing to the fraction of oscillatory responses observed on negative control and weakly activating coverslips (Supplementary Fig. S1). These results indicate the importance of characterizing the levels of Ca^{2+} in the cellular environment when conducting Ca^{2+} response experiments.

V. SUPPLEMENTARY TABLE

Antigen	k_{off} rate	Reference
UCHT1	0.01	(Salmerón, A. et al., 1991)[30]
9V:MHC	0.33	(Aleksic, M et al., 2010)[23]
OKT3	0.39	(Kjer-Nielsen, L et al, 2004)[29]
4D:MHC	2.59	(Aleksic, M et al., 2010)[23]

Table S1: The k_{off} rate of each antigen and corresponding references.

VI. SUPPLEMENTARY FIGURES

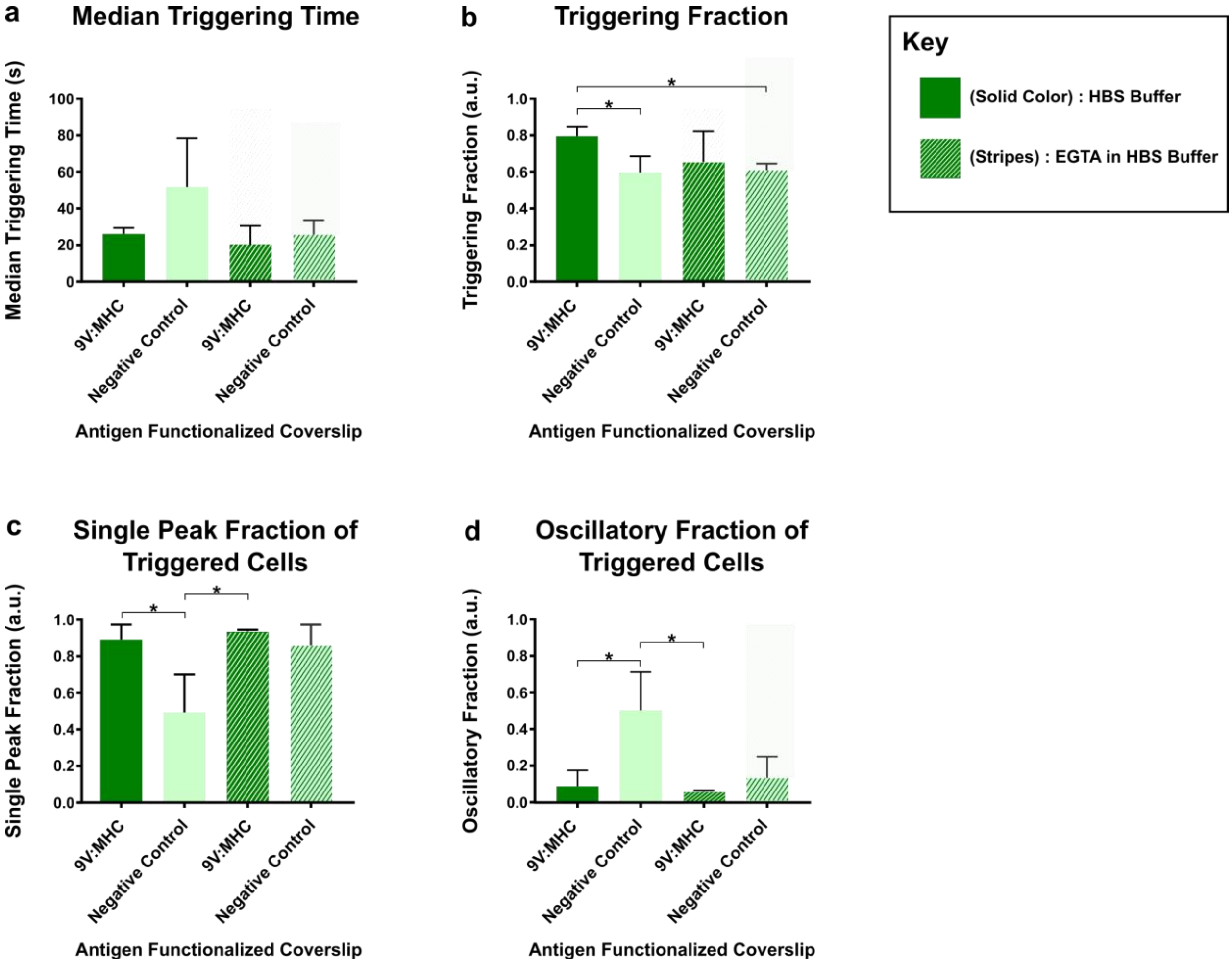

Fig. S1: Calcium patterns in T cells when interacting with antigen functionalized coverslip in an HBS buffer or an EGTA in HBS buffer. T cells were stimulated by the antigen, 9V:MHC, or by no specific antigen, negative control in either an HBS buffer or an HBS buffer supplemented with EGTA. N = 100-1000. (a) The median triggering time, the time between landing and the first calcium flux. (b) The triggering fraction. (c) The triggered T cells' single peak fraction. (d) The triggered T cells' oscillatory fraction.

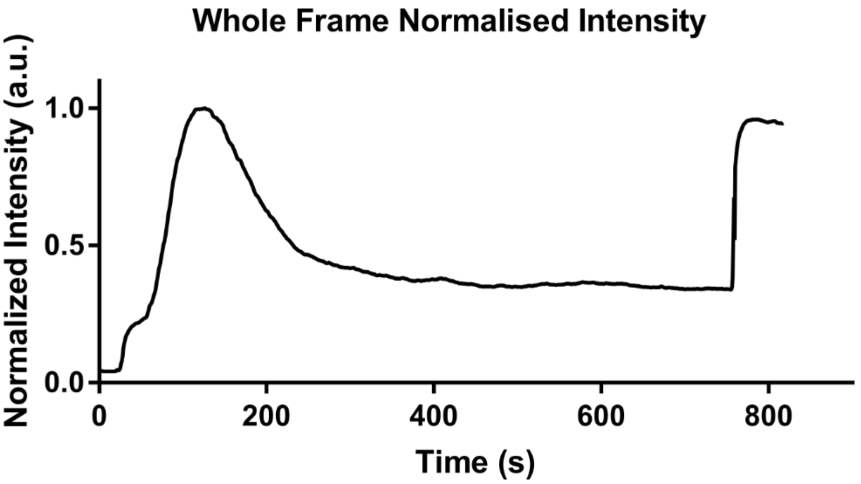

Fig. S2: Integrated fluorescence intensity from the whole field of view for Jurkat T-cells loaded with Fluo4-AM exposed to glass coated with 4D:MHC showing minimal fluorescence loss due to photo-bleaching on the time scale of calcium dynamics.

VII. SUPPLEMENTARY MOVIES

Supplementary Movie S1. Time-lapse images of Ca^{2+} dependent fluorescence, using Fluo-4 AM (maximum projection over 1000 frames), detected in 1G4-TCR Jurkat T-cells when landing on a coverslip functionalized with **UCHT1**, a αCD3 antibody. Imaging includes cells pipetted onto the coverslip and the addition of ionomycin in the last 100 frames. Scale bar: 100 µm. Frame time: 0.82 s. Total duration: ~13.5 min.

Supplementary Movie S2. Time-lapse images of Ca^{2+} dependent fluorescence, using Fluo-4 AM (maximum projection over 1000 frames), detected in 1G4-TCR Jurkat T-cells when landing on a coverslip functionalized with **9V:MHC**, a peptide presented on MHC Class I. Imaging includes cells pipetted onto the coverslip and the addition of ionomycin in the last 100 frames. Scale bar: 100 µm. Frame time: 0.82 s. Total duration: ~13.5 min.

Supplementary Movie S3. Time-lapse images of Ca^{2+} dependent fluorescence, using Fluo-4 AM (maximum projection over 1000 frames), detected in 1G4-TCR Jurkat T-cells when landing on a coverslip functionalized with **OKT3**, a αCD3 antibody. Imaging includes cells pipetted onto the coverslip and the addition of ionomycin in the last 100 frames. Scale bar: 100 µm. Frame time: 0.82 s. Total duration: ~13.5 min.

Supplementary Movie S4. Time-lapse images of Ca^{2+} dependent fluorescence, using Fluo-4 AM (maximum projection over 1000 frames), detected in 1G4-TCR Jurkat T-cells when landing on a coverslip functionalized with **4D:MHC**, a peptide presented on MHC Class I. Imaging includes cells pipetted onto the coverslip and the addition of ionomycin in the last 100 frames. Scale bar: 100 µm. Frame time: 0.82 s. Total duration: ~13.5 min.

Supplementary Movie S5. Time-lapse images of Ca^{2+} dependent fluorescence, using Fluo-4 AM (maximum projection over 1000 frames), detected in 1G4-TCR Jurkat T-cells when landing on a coverslip without a specific antigen functionalized (**Negative control**). Imaging includes cells pipetted onto the coverslip and the addition of ionomycin in the last 100 frames. Scale bar: 100 µm. Frame time: 0.82 s. Total duration: ~13.5 min.
